# A reversible posterior leucoencephalopathy syndrome including blindness caused by preeclampsia

**Published:** 2016-09

**Authors:** G Vandenbossche, J Maquet, P Vroonen, G Lambert, M Nisolle, F Kridelka, E Emonts

**Affiliations:** CHU of Liège, Department of Obstetrics and Gynaecology, 4000 Liège, Belgium.; University of Liège, Medical School, Liege, Belgium.; CHU of Liège, Department of Emergencies, Liège, Belgium.; CHR de la Citadelle, Department of Anesthesiology, 4000 Liège, Belgium.; CHR de la Citadelle, Department of Obstetrics and Gynecology, Liège, Belgium.

**Keywords:** Blindness, posterior reversible encephalopathy syndrome, PRES, preeclampsia, pregnancy complication

## Abstract

Complications of (pre)eclampsia may involve multiple systems and organs. Neurological symptoms may occur. Visual symptoms concern up to 25% the of patients with severe preeclampsia and 50% of the patients with eclampsia. An uncommon effect of severe preeclampsia is sudden blindness. Blindness may be part of a clinical and radiological presentation named Posterior Reversible Encephalopathy Syndrome (PRES). PRES may lead to permanent neurological deficit, recurrences or death. We report the case of a 24-year-old Caucasian patient, gravida 5 para 2 who developed preeclampsia and PRES complicated with blindness at 32 weeks of gestation. Optimal care allowed visual symptoms to resolve within 24 hours and a favourable maternal outcome and no long- term sequelae. We describe different causes and manifestations of PRES and highlight the need for immediate care in order to optimize the chance of symptoms reversibility.

## Introduction

Preeclampsia affects 3–5% of pregnant patients and is a leading cause of maternal and foetal morbidity- mortality, particularly in developing countries (Walker, 2000). This multisystem disorder can include cardiovascular changes, hematologic abnormalities, hepatic and renal impairment and neurologic manifestations (Williams et al., 2011). Visual pathways may also be affected (Roos, 2012). Visual symptoms concern up to 25% of the patients with severe preeclampsia and 50% of the patients with eclampsia (Sunness et al., 1988).

Posterior Reversible Encephalopathy Syndrome (PRES) is a clinical and radiological neurological syndrome described in 1980 by Hynchey et al. (1996). PRES may develop in the context of renal failure, immunosuppressive therapy, porphyria, high blood pressure, hypertensive encephalopathy, preeclampsia and eclampsia (Hinchey et al., 1996; Onder et al., 2007).

Physiology of PRES is not completely understood but hypertension and vasogenic oedema secondary to increased capillary permeability are often cited (Wagner, 2011; Staykov, 2013).

PRES associates seizure activity, consciousness impairment, headache, nausea and focal neurological signs (Hinchey et al., 1996). Visual abnormalities are also described with rarely cortical blindness (Cunningham et al., 1995).

PRES may be reversible if adequate and timely treatment is initiated but may be permanent, recurrent or lead to a fatal outcome if optimal care is delayed (Pizon et al., 2015). No clinical trials have evaluated the management of PRES, but rapid withdrawal of the trigger appears to hasten recovery and to avoid complications such as aggressive blood pressure management and withdrawal of the offending drug (Roth et al., 2011).

Cerebral imaging abnormalities are often symmetric and predominant in the posterior white matter (Peng et al., 2008). Oedema is frequently reported at computed tomography (CT) and at magnetic resonance imaging (MRI) (Doelken et al., 2007).

We report the case of a 24-year-old patient with clinical and radiological presentation of PRES complicated by blindness.

Our aim is to emphasize the critical importance of early diagnosis and immediate care in order to avoid long term or permanent complications.

## Case report

A 24-year-old, Caucasian woman, gravida 5 para 2 presented at the Obstetrical Emergencies at 32 weeks gestation complaining of headache, abdominal pain and absence of foetal movements. Regular follow-up of the on-going pregnancy had, so far, been uneventful. Before the event, the patient has no symptoms, no on-going oedema, no hypertension and no visual disturbances. Past medical history was unremarkable.

At the time of admission, blood pressure was 180/120 mm Hg. No foetal heart activity was noted at cardiotocogram and ultrasound. Intrauterine foetal death at 32 weeks of gestation was confirmed.

On physical examination, no peripheral oedema was present. A Bishop score of 4 was noted.

Laboratory tests revealed proteinuria (2 +), a mild elevation of uric acid (7mg/dL) and LDH (750 UI/L). Hepatic tests and platelets counts were normal.

A diagnosis of severe preeclampsia complicated with foetal death was confirmed.

Urgent labour induction was advised and antihypertensive treatment was initiated without delay with partial correction of hypertension (150/110 mm Hg) by oral nifedipine. Blood pressure was continuously monitored.

One hour after admission, the patient complained of sudden bilateral visual loss. Blood pressure had peaked at 190/120 mm Hg. IV antihypertensive was immediately adapted (nicardipine) and magnesium sulphate (4g bolus and then 1g/h by continuous infusion) was given. Partial blood pressure reduction to 165/95 mm Hg was obtained.

The patient was counselled on the need to realize an emergency caesarean section with the delivery of a dead female newborn of 1710 g.

Subsequent neuro/ophthalmological examination of the mother revealed brisk reflexes and bilateral papilloedematous discs with macular oedema.

**Fig. 1:**
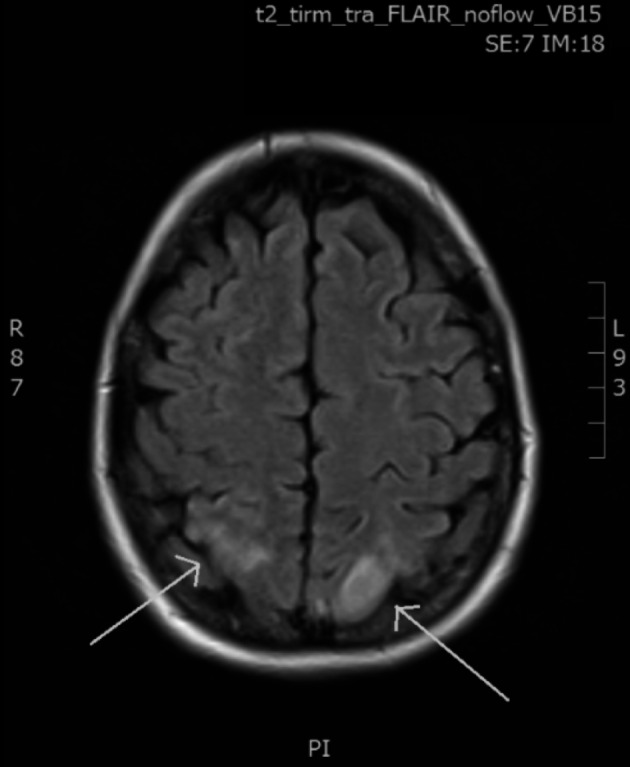
— MRI performed during episode of blindness: posterior lesions in both occipital poles with a hyperintense signal (green arrow) on FLAIR sequence.

Brain CT-Scan showed a low-density lesion in the right parietal pole. The electroencephalogram showed signs of bilateral occipital suffering. The magnetic resonance imaging, known to be more accurate in such conditions, diagnosed posterior focal lesions in both occipital poles with a hyperintense signal on fluid attenuated inversion recovery (FLAIR) sequence (Fig. 1.).

A diagnosis of PRES syndrome including acute blindness caused by preeclampsia was confirmed.

Continuous Intravenous antihypertensive therapy (nicardipine) was delivered over the next three days under intensive surveillance in the IMC (Intensive Maternal Care) department with the aim to optimally equilibrate the patient’s blood pressure. The patient regained vision in both eyes within the first 24 hours. Blood pressure was definitely normalised after three days of antihypertensive treatment.

A control MRI and electroencephalogram were performed two weeks after the caesarean section. Both showed a near complete regression of the cerebral lesions.

Renal function remained stable within one month after delivery. No hypertension persisted.

The following year, the patient gave birth to a live male infant of 3240 g at 37 weeks after an uncomplicated pregnancy. The patient received low- dose aspirin from the beginning of her pregnancy until the 36th week of gestation. An elective caesarean section was performed.

## Discussion

PRES (Posterior Reversible Encephalopathy Syndrome) refers to several pathological conditions that share the same clinical and radiological presentations and result in the development of neurological deficit (Hinchey et al., 1996).

The prevalence of this complication is unknown. There is a marked female predominance that may reflect some of the underlying causes. Hypertension is the second most common associated condition after toxic agents (cancer chemotherapy agents such as cysplatin, anti-angiogenic agents such as Bevacizumab,... ), the latter being present in 6% to 72 % of cases (Bartynski et al., 2006; Lee et al., 2008). Preeclampsia is found in 7% to 20% of patient with PRES (Hinchey et al., 1996; Schwartz, 2000).

The precise pathophysiology of PRES remains unclear. Cerebral vasospasm with secondary ischemic injury or vasogenic oedema due to increased capillary permeability are both suggested (Servillo et al., 2007; Staykov et al., 2013). Recently, single-photon emission tomography studies supported the finding that elevated capillary permeability and vasogenic oedema are present in patients with cortical blindness (McCormick et al., 1999).

Despite the dramatic neurological and radiological picture, the syndrome may be reversible, as suggested by its name (Schwartz, 2002). The rate of reversibility varies widely in published series (from 35% to 100%). Reversibility, whether complete or partial, is not always mentioned in the literature (Hinchey, 1996). Among patients with a follow-up CT or MRI, 49% to 75% have a resolution of the initial abnormalities within 5 days to 17 months (Casey et al., 2010). However, if not adequately and quickly treated, there may remain permanent neurological deficits, most frequently under the form of epilepsy (Burnett et al., 2010).

Permanent neurological abnormalities are related to ischemia and/or bleeding. Death is reported in up to 15% of the patients with PRES (Servillo et al., 2003, Lee, 2008; Striano et al., 2010). Delayed identification or care of the patient appears to be involved in most patients with a fatal outcome.

The combination of suggestive clinical manifestations and typical radiological criteria confirm the diagnosis of PRES. Neurological manifestations such as headache, nausea, vomiting, confusion, behavioural disorders, impaired level of consciousness (from somnolence to coma), mental status changes (confusion, amnesia, impaired concentration, lethargy), and seizures may occur (Hinchey et al., 1996).

Visual abnormalities (blurred vision, hemianopsia...) are found in 26 % to 67% (Hinchey et al., 1996; Bartynski et al., 2005) of the patients with PRES and cortical blindness in 8% to 33 % (Burnett et al., 2010). In the past, most cases of blindness in preeclampsia and eclampsia were commonly attributed to retinal pathology. The latter include vascular abnormalities, oedema or detachment and acute ischemic optic neuropathy as a result of decreased blood supply to the prelaminar portion of the optic nerve. Nowadays, more emphasis is being placed on the cortical aetiology of blindness (Schultz et al., 2005).

Diagnostic strategy for PRES must be standardized. After clinical history, physical examination should be immediately followed by imaging and blood sample. MRI has a sensitivity superior to that of CT (Doelken et al., 2007). Laboratory tests should be obtained routinely (Gao et al., 2012). Electroencephalography (EEG) has to be performed to investigate non-convulsive status epilepticus. An early etiologic diagnosis allows prompt correction of the cause of PRES.

Control of hypertension is a crucial part of the symptomatic management. Until better evidence is available, the choice of antihypertensive should depend on the clinician’s experience and familiarity with a particular drug. Exceptions are nimodipine, magnesium sulphate (although this is indicated for women who require an anticonvulsant for prevention or treatment of eclampsia), diazoxide and ketanserin, which are probably best avoided (Duley et al., 2013). Treatment is recommended for SBP ≥ 160 mmHg and/or DBP ≥ 110 mmHg with a target between 130 and 150 mmHg for SBP, and between 80 and 100 mmHg for DBP (Vaughan et al., 2010; Ramsay et al., 1999). Immediate and acute blood pressure reduction can aggravate the cerebral perfusion pressure alterations and promote ischemia (Varon et al., 2010). A decrease of 10 to 20 mmHg every 10 to 20 min has been suggested by some authors (Dennis et al., 2012). Appropriate medications include nicardipine and labetolol (Varon et al., 2010). The literature is not clear whether nifedipine can increase angio-oedema and triggered the PRESS. Nicardipine has higher selectivity for blood vessels than the myocardium and causes less reflex tachycardia than nifedipine. Furthermore, the dosage of nicardipine can be more easily adjusted. The use of thiopental, valproate or phenitoin was reported only for status epilepticus (Demirel et al., 2012).

Correction of the cause is crucial in order to decrease the risk of ischemia or bleeding and to avoid permanent disability or death.

The only etiologic treatment of preeclampsia is foetus and placenta delivery. Timing of delivery must take into account the gestational age, severity of preeclampsia, as well as maternal and foetal conditions. Current treatments aim at avoiding maternal complications such as cerebral haemorrhage, pulmonary oedema, and eclampsia. Treatment is essentially based on antihypertensive therapy and magnesium sulphate (MgSO4).

MgSO4 is the cornerstone of the prevention and treatment of eclampsia. Its use is associated with a 50% reduction of eclampsia episodes in severe preeclampsia. It also reduces the risk of maternal death. The number needed to treat (NNT) to observe 1 beneficial effect is 50 for severe preeclampsia patients, whereas it rises to 100 for patients with preeclampsia without severe features (Duley et al., 2010). In case of severe preeclampsia arising in the postpartum period, the administration of MgSO4 is also recommended for at least 24 hours. The effect of MgSO4 is likely multifactorial, including both vascular and neurological mechanisms. Magnesium is a calcium antagonist and induces vasodilation. In addition, MgSO4 may decrease blood brain barrier (BBB) permeability and limit vasogenic oedema (Euser et al., 2008). In addition, MgSO4 has anticonvulsant properties, which may be related to its N-methyl-D-aspartate (NMDA) glutamate receptor antagonist activity.

Pulmonary oedema is a potential complication of preeclampsia. Decreased colloid osmotic pressure, increased capillary permeability, increased hydrostatic pressure and cardiac diastolic dysfunction may all contribute to this complication. Preeclampsia is also regarded as a hemodynamic state of depleted intravascular volume, submitting the patient to a higher risk of renal failure. The intravenous administration of fluids to increase plasma volume or to improve renal perfusion is not recommended in women with normal renal function. In case of oliguria, variable invasive monitoring has been proposed to guide fluid therapy. This invasive monitoring may be associated with several complications. Echocardiography and pulmonary ultrasound, allowing interstitial fluid imaging (B lines), may provide useful information to guide fluid therapy in this situation, where the risk of renal failure must be balanced against the risk of pulmonary oedema (Euser et al., 2008).

Current guidelines for obstetricians and midwives support the use of low-dose aspirin for the prevention of preeclampsia in women at risk of this condition. However, guidance regarding what level of preeclampsia risk warrants aspirin prophylaxis, is not clear yet. This uncertainty has resulted in inconsistencies in recommendations and possible underuse of aspirin. Without an agreement upon threshold of risk for PE, based upon valid algorithms, it is problematic for clinicians to identify which women should receive aspirin.

## Conclusion

We report a case of reversible posterior encephalopathy syndrome (PRES) secondary to preeclampsia complicated by sudden blindness. The patient regained vision in both eyes and had a favourable outcome with no sequelae after appropriate treatment.

This case highlights the utmost importance of an early diagnosis followed by immediate and appropriate care. Both parameters optimize the chance of complete reversibility and limit the risk of neurological sequelae or potential fatal evolution.

Our observation, combined with other reported cases, leads some authors to suggest that a better name may be “potentially reversible encephalopathy syndrome”.
